# Structure and optical properties of perovskite-embedded dual-phase microcrystals synthesized by sonochemistry

**DOI:** 10.1038/s42004-020-0265-6

**Published:** 2020-02-07

**Authors:** Sangyeon Cho, Seok Hyun Yun

**Affiliations:** 1 Wellman Center for Photomedicine, Massachusetts General Hospital and Harvard Medical School, Cambridge, MA 02139 USA; 2 Harvard-MIT Health Sciences and Technology, Massachusetts Institute of Technology, Cambridge, MA 02139 USA

**Keywords:** Optical materials, Semiconductor lasers

## Abstract

Cesium lead halide perovskite (CsPbX_3_, X=Cl, Br, I) nanocrystals embedded in Cs_4_PbX_6_ or CsPb_2_X_5_ matrices have received interests due to their excellent optical properties. However, their precise endotaxial structures are not known, and the origin of photoluminescence remains controversial. Here we report a sonochemistry technique that allowed us to synthesize high-quality CsPbBr_3_-based microcrystals in all ternary phases, simply by adjusting precursor concentrations in a polar aprotic solvent, N,N-dimethylformamide. The microcrystals with diverse morphologies enabled us to visualize the lattice alignments in the dual-phase composites and confirm CsPbBr_3_ nanocrystals being the photoluminescent sites. We demonstrate high solid-state quantum yield of >40% in Cs_4_PbBr_6_/CsPbBr_3_ and lasing of CsPbBr_3_ microcrystals as small as 2 µm in size. Real-time optical analysis of the reaction solutions provides insights into the formation and phase transformation of different CsPbBr_3_-based microcrystals.

## Abstract (web summary)

The origin of the excellent photoluminescence properties of embedded cesium lead halide perovskite nanocrystals remains to be fully understood. Here the authors visualize lattice alignments in dual-phase Cs_4_PbBr_6_ and CsPb_2_Br_5_ composites synthesized by sonochemistry and find that the CsPbBr_3_ nanocrystals are responsible for the luminescence.

## Introduction

Three-dimensional (3D) lead halide perovskites (LHPs) with the form of APbX_3_ (A=Cs^+^, CH_3_NH_3_^+^, X=Cl^−^, Br^−^, I^−^) are promising optical materials. These materials offer a long carrier lifetime (>1 μs), long exciton diffusion length^1,2^ (>1 μm), large optical cross-sections^3^ (~10^−13^ cm^2^), and defect tolerance owing to the antibonding character of the conduction and valence bands^4^. These properties make them an attractive building block for solar cells, light-emitting diodes, and lasers^5^. Among various types of LHPs, all-inorganic CsPbBr_3_ received increasing interest due to their high luminescent quantum yields in solid states in the green range and superior environmental stability to other perovskites with organic cations. CsPbBr_3_ has two lower-dimensional counterparts: zero-dimensional (0D) Cs_4_PbBr_6_ and quasi-two-dimensional (2D) CsPb_2_Br_5_. They represent different ternary phases of the Cs–Pb–Br compounds and can be formed from precursors, such as CsBr and PbBr_2_ (Supplementary Fig. 1)^6^. Cs_4_PbBr_6_ and CsPb_2_Br_5_ are known to have large bandgap energies of 3.7 eV and 3.1 eV, respectively^7,8^. Nonetheless, some confusion has arisen when these non-perovskite materials were claimed to generate green photoemission^4,9^. Recent studies^7,10–12^ have suggested that these materials contain CsPbBr_3_ nanocrystals (NCs) that are responsible for photoluminescence.

We refer these dual-phase materials to as Cs_4_PbBr_6_/CsPbBr_3_ (CsPbBr_3_ NCs in a Cs_4_PbBr_6_ matrix) and CsPb_2_Br_5_/CsPbBr_3_ (CsPbBr_3_ NCs in a CsPb_2_Br_5_ matrix). While much progress in the dual-phase materials is expected, it has been difficult to visualize the relative lattice orientation between LHPs NCs and the host matrix clearly by using HRTEM, due to difficulties such as the low damage threshold of the materials by electron beam and the inadequate sample sizes being too small lateral size (<10 nm) to observe more than four different lattice planes at given electron beam or too large in thickness (larger than few hundreds of nm) in thickness to get clear images^7,12–14^.

Herein, we report a new method based on sonochemistry that enables a facile, rapid synthesis of various phase, and dimensional CsPbBr_3_ perovskites microcrystals in a polar aprotic solvent, N,N-dimethylformamide. We show this technique can produce both types of dual-phase materials, as well as single-phase LHP microcrystals, with various surface morphologies depending on precursor concentrations. Our investigation provides insights into the formation kinetics and phase transition of the Cs–Pb–Br compounds. The produced microparticles enabled us to investigate the lattice structures and optical properties of the various CsPbBr_3_-based compounds.

## Results and discussion

### Sonochemical synthesis of various LHPs

**Fig. 1:**
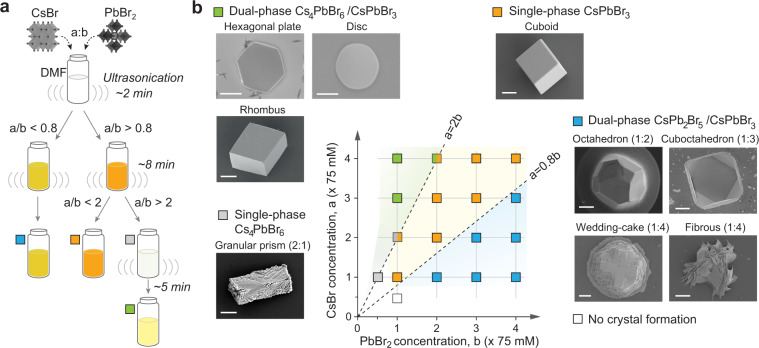
Sonochemical synthesis of various CsPbBr_3_-based microcrystals. **a** Schematic of the sonochemical synthesis using ultrasonication of CsBr and PbBr_2_ salts in DMF. The starting concentration of the precursor materials are denoted as “*a*” and “*b*”. **b** Two-dimensional concentration phase diagram of the sonochemical reaction products. SEM images of dual-phase Cs_4_PbBr_6_/CsPbBr_3_ (hexagonal plate, microdisc, and rhombus), single-phase Cs_4_PbBr_6_ (granular prism), single-phase CsPbBr_3_ (cuboid), dual-phase CsPb_2_Br_5_/CsPbBr_3_ (truncated octahedron, cuboctahedron, wedding cake, and fibrous). Scale bars, 2 μm.

The general scheme of sonochemical synthesis starts with placing two precursor salts, CsBr and PbBr_2_, with sufficient quantities beyond their maximum soluble amount in a polar aprotic solvent. For CsBr and PbBr_2_ salts, N,N-dimethylformamide (DMF) produced high-quality CsPbBr_3_ microparticles among different polar aprotic solvents having similar dipole moments (acetone, ethyl acetate (EtoAC), γ-butyrolactone (GBL), and dimethyl sulfoxide (DMSO)) (Supplementary Fig. 2). So, all the experiments presented herein after were obtained using DMF. As illustrated in Fig. 1a, after several minutes of ultrasonication at room temperature (Supplementary Fig. 3), the salts are fully dissolved and produce a stable solution. The final solution varies in color, depending on the ratio of the starting concentration of the precursors (Supplementary Fig. 4).

Figure 1b summarizes our finding arranged in a two-dimensional phase diagram, along with the SEM images of various distinct types of microparticles formed. We used parameters “*a*” and “*b*” to denote the concentrations of the CsBr and PbBr_2_ precursors, respectively. The values are normalized to 75 mM (e.g., “*a* = 1” corresponds to 75 mM of CsBr, and “*b* = 2” refers to 150 mM of PbBr_2_).

From stoichiometry, the ideal concentration ratio to produce CsPbBr_3_ would be *a*/*b* = 1, if the two precursor materials were equally dissolved in the solvent. Experimentally, CsPbBr_3_ microcrystals were produced when the ratio a/b was approximately between 0.8 and 2, and both “*a*” and “*b*” are in a range from 1 to 4 (i.e., 75–300 mM). The orange-color solution obtained after ultrasonication contains single-phase CsPbBr_3_ microparticles with the cuboidal shape (Supplementary Fig. 5).

When *a*/*b* <0.8 and *b* >1, the product of reaction is dual-phase CsPb_2_Br_5_/CsPbBr_3_ composites, which show intense yellow color under room light. Their surface morphology varied depending on the precursor concentration (Supplementary Fig. 6). For *a* = 1 (75 mM) and *b* = 2 or 3 (75 or 150 mM), the microparticles have largely octahedral shapes. With *b* = 4 (300 mM), the particles tend to have irregular shapes and rough surfaces, probably resulting from rapid surface nucleation due to the high PbBr_2_ concentration. No crystals were formed at low concentrations of *a* = 0.5 (37.5 mM) and *b* = 1 (75 mM).

When *a*/*b* >2 and *a* >2 (150 mM), we found that single-phase CsPbBr_3_ microcrystals are initially formed, but converted to white-color Cs_4_PbBr_6_ microcrystals and then to lemon-color dual-phase Cs_4_PbBr_6_/CsPbBr_3_ composites. Cs_4_PbBr_6_/CsPbBr_3_ composites are found in a mixture of rhombus and hexagonal plates (Supplementary Fig. 7). When these Cs_4_PbBr_6_/CsPbBr_3_ plates are left for about 1 h in the solution with ultrasonication being off, they undergo phase transition to Cs_4_PbBr_6_/CsPbBr_3_ with micro-discoidal shapes. The same morphological transition was observed when the solution was vigorously shaken by hands. This dynamic process is described later in more detail.

When *a*/*b* = 2 and *a* <= 2 (150 mM), CsPbBr_3_ microparticles are initially formed and converted to Cs_4_PbBr_6_ microcrystals, and they remain as the final product. Single-phase Cs_4_PbBr_6_ microcrystals have a granular structure (Supplementary Fig. 8).

### Structures of dual-phase LHPs

To identify the crystal structure and stoichiometry of the various products, we performed powder X-ray diffraction (PXRD) and energy dispersive X-ray spectroscopy (EDS). The data (Supplementary Figs. 9–12) confirmed the orthorhombic structure of CsPbBr_3_ (space group *Pbnm*, *A* = 8.20 Å, *B* = 8.24 Å, *C* = 11.74 Å), the trigonal structure of Cs_4_PbBr_6_ in the single- and dual-phase Cs_4_PbBr_6_ products (*R*$\bar 3$*c*, *A* = *B* = 13.73 Å, *C* = 17.32 Å), and the tetragonal structure of CsPb_2_Br_5_ in the dual-phase composite (*I4/mcm*, *A* = *B* = 8.45 Å, *C* = 15.07 Å). These results are consistent with previous reports^8,15,16^.

Unlike previous dual-phase materials^7,12–14^, structurally anisotropic microparticles produced by sonochemistry were well suited to obtain high-quality HRTEM having more than four different lattice planes at given the direction of an electron beam. For HRTEM imaging, we used micro-discoidal Cs_4_PbBr_6_/CsPbBr_3_ (*a* = 3 (225 mM), *b* = 1 (75 mM)) having average thickness of 80 nm (*N* = 15) and wedding-cake CsPb_2_Br_5_/CsPbBr_3_ (*a* = 1 (75 mM), *b* = 4 (300 mM)) consisting of multiple thin layers having average thickness of 50 nm (*N* = 15) (Supplementary Fig. 13).

**Fig. 2:**
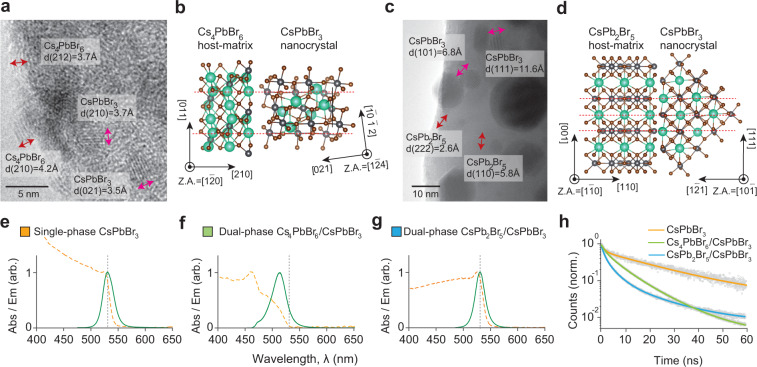
Structural and optical characterization of the CsPbBr_3_-based materials. **a** HRTEM image of a dual-phase Cs_4_PbBr_6_/CsPbBr_3_ microdisc. **b** Cs_4_PbBr_6_ and CsPbBr_3_ crystal structure based on the HRTEM image. **c** HRTEM image of a dual-phase CsPb_2_Br_5_/CsPbBr_3_ wedding-cake crystal. **d** CsPb_2_Br_5_ and CsPbBr_3_ crystal structure based on the HRTEM image. **e**–**g** Absorbance (dashed orange lines) and fluorescence (solid green lines; excitation at 480 nm) spectra of CsPbBr_3_, Cs_4_PbBr_6_/CsPbBr_3_, and CsPb_2_Br_5_/CsPbBr_3_. **h** Time-resolved photoluminescence measurement. The measured data (circles) are fitted with triple exponential curves (lines).

The real-space images and the corresponding fast Fourier transform (FFT) analysis revealed the relative lattice orientation of the endotaxial structures (Fig. 2a, c; Supplementary Fig. 14). In the case of Cs_4_PbBr_6_/CsPbBr_3_, well-defined lattice fringes with 3.7 Å and 4.2 Å having intersection angle of 42° are indexed to (212) and (210) of the Cs_4_PbBr_6_ matrix, and higher-contrast lattice fringes with 3.7 Å and 3.5 Å having intersection angle of 67° are indexed to (210) and (021) of the CsPbBr_3_ NCs. This suggests that the [210] axis of Cs_4_PbBr_6_ is tilted by 6° with respect to the [021] axis of CsPbBr_3_. For CsPb_2_Br_5_/CsPbBr_3_, low-contrast lattice fringes with 2.6 Å and 5.8 Å having intersection angle of 35° are indexed to (222) and (110) of the CsPb_2_Br_5_ matrix, and lattice fringes in darker sub-regions with 6.8 Å and 11.6 Å having intersection angle of 35° are indexed to (210) and (021) of the CsPbBr_3_ NCs. Hence, the [110] axis of CsPb_2_Br_5_ is aligned to the [1$\bar 2$1] axis of CsPbBr_3._ A computational model based on the data confirmed good facet matching between CsPbBr_3_ NCs and non-perovskite matrices (Fig. 2b, d). From the TEM images, we determined the effective size of CsPbBr_3_ NCs embedded in the matrices by measuring the diameter of the largest circle circumscribing the NCs (Supplementary Fig. 15). The CsPbBr_3_ NCs in Cs_4_PbBr_6_ have sizes of 3–5 nm with a mean effective diameter of 4.2 nm. The CsPbBr_3_ NCs in CsPb_2_Br_5_ are larger, ranging from 10 to 20 nm, with a mean effective diameter of 14.6 nm.

### Optical properties of dual-phase LHPs

Using a custom-built microscope coupled with a grating-based spectrometer (Supplementary Fig. 16), we measured the optical emission and absorption spectra of various product particles either in solution (Fig. 2e–g). The optical spectra did not change after the particles have been transferred to a glass substrate. CsPbBr_3_ microcrystals have an absorption edge at 538 nm (2.31 eV), weak excitonic peak at 523 nm, and low Urbach energy of 23 meV. Dual-phase Cs_4_PbBr_6_/CsPbBr_3_ microcrystals have an absorption edge at 525 nm (2.37 eV), and their fluorescence peaks are blue-shifted to 512 nm. The magnitude of blue shift varied between 10 and 21 nm, depending on the precursor ratio and the conversion method (Supplementary Fig. 17). The bandgap changes, *ΔE*, by quantum confinement is given by:

1 $$\Delta E \approx \frac{{{\hbar} ^2\pi ^2}}{{2d^2}}\left( {\frac{1}{{m_e^ {\ast} }} + \frac{1}{{m_h^ {\ast} }}} \right) - \frac{{1.8\,e^2}}{{4\pi \varepsilon d}}$$

where $m_h^ \ast = 0.14$ and $m_e^ \ast = 0.15$ denote the effective mass of the hole and electron^17^, respectively, in CsPbBr_3_ in the unit of the electronic mass, *d* the diameter of a spherical potential well, ε the permittivity of the matrix surrounding CsPbBr_3_ NCs. CsPbBr_3_ NCs. *ε/ε*_*0*_ = 3.1 was calculated for Cs_4_PbBr_6_ by a density functional theory^18^. The spectral shifts we measured from the spectra indicate *d* = 6.2 nm and 5.6 nm, respectively. These values are reasonable, but larger than the mean diameter of 4.2 nm. The discrepancy may be attributed to the nonspherical shapes of the nanocrystals and interfacial effects with the Cs_4_PbBr_6_ matrix. The dielectric constant of CsPb_2_Br_5_ matrix is unknown. Assuming it is the same as Cs_4_PbBr_6_, the quantum confinement effect for NCs with the mean diameter of 14.6 nm is estimated to be −6 meV. The fluorescence peak of CsPb_2_Br_5_/CsPbBr_3_ microparticles is at 530 nm, ~1 nm shifted from the 531 nm peak of CsPbBr_3_ microcrystals. This shift of ~4.4 meV corresponds to *d* = 11 nm.

We investigated time-resolved photoluminescence using a picosecond frequency-doubled laser (*λ* = 382 nm). The experimental time-resolved photoluminescence data (Fig. 2h; Supplementary Table 1) were fitted to a three-exponential decay curve:

2 $$f\left( t \right) = A_1e^{ - \frac{t}{{\tau _1}}} + A_2e^{ - \frac{t}{{\tau _2}}} + A_3e^{ - \frac{t}{{\tau _3}}}$$

where *A*_1_, *A*_2_, and *A*_3_ are pre-exponential factors, and *τ*_1_, *τ*_2_, and *τ*_3_ are lifetime constants. The total decay time was computed from weighted lifetime constants:

3 $$\tau _{{\mathrm{tot}}} = A_1\tau _1 + A_2\tau _2 + A_3\tau _3$$

The radiative decay time of the sample is related to total decay time and absolute PLQY:

4 $$\tau _{{\mathrm{rad}}} = \tau _{{\mathrm{tot}}} \ast {\mathrm{PLQY}}$$

Dual-phase Cs_4_PbBr_6_/CsPbBr_3_ has a much faster radiative lifetime of 9.7 ns, compared with the lifetime of 1.2 μs for single-phase CsPbBr_3_ and 4.4 µs for dual-phase CsPb_2_Br_5_/CsPbBr_3_. Cs_4_PbBr_6_/CsPbBr_3_ has a high photoluminescence quantum yield (PLQY) of over 40% due to both the quantum confinement and low dielectric constant of Cs_4_PbBr_6_^13^.

### Lasing of optically pumped LHP microcrystals

**Fig. 3:**
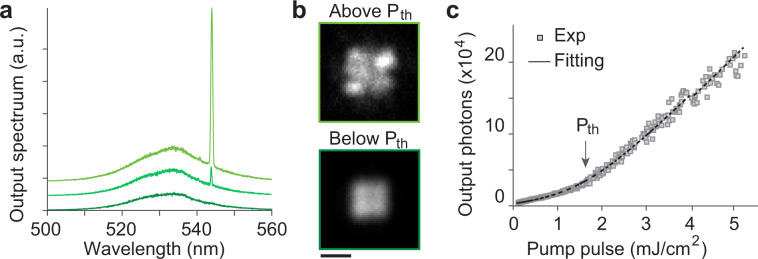
Lasing from single-phase CsPbBr_3_ microcrystals. **a** Output spectra from a 2-μm sized CsPbBr_3_ microcrystal upon nanosecond optical pumping (480 nm) below and above lasing threshold. **b** Wide-field fluorescence images below and above laser threshold. Scale bar, 2 μm. **c** A light-in–light-out curve, showing a threshold pump fluence of 1.7 mJ/cm^2^ and a spontaneous emission factor (β) of ~0.05.

Single-mode lasing from single-phase CsPbBr_3_ microcrystals was observed when excited by nanosecond-pulsed optical pumping at 480 nm (Fig. 3; Supplementary Fig. 18). The smallest size of lasing CsPbBr_3_ microcrystals was 2 µm (Fig. 3b; Supplementary Fig. 18). The laser emission linewidth was ~0.2 nm above a threshold pump energy of 1.7 mJ/cm^2^, and the spontaneous emission factor (β) was 0.05 (Fig. 3c). The other device presents the laser emission linewidth of 0.3 nm above a threshold pump energy of 2.2 mJ/cm^2^, and the spontaneous emission factor was 10^−3^ (Supplementary Fig. 18). The converted threshold pump energy to threshold carrier density is ~3 × 10^19^ cm^−3^, which is higher than theoretical estimation of Mott density (10^18^ cm^−3^)^19^. This infers lasing in the electron hole plasma state (EHP), rather than excitonic state^5,19^, which is beneficial to build up large population inversion via bandgap renormalization (BGR)^20^. The laser emission of single-phase CsPbBr_3_ microparticles in air (15 samples) at a pump fluence twice the lasing threshold was prolonged for 10^5^ pump pulses (5000 s at 20 Hz) with a pulse-to-pulse wavelength fluctuation of 0.47 nm (Supplementary Fig. 18). On the contrary, dual-phase microparticles did not support laser oscillation even at higher pump energy levels up to tens of mJ/cm^2^. Considering the high PLQY of CsPbBr_3_ NCs in the dual-phase composites, we attribute the failure to reach lasing threshold to the weaker cavity resonance due to the lower refractive index (~1.8) of the Cs_4_PbBr_6_^21^ and CsPb_2_Br_5_ matrices compared with the index (~2.6) of CsPbBr_3_^5^, and relatively small amount of the optical gain in the excitonic state due to un-normalized bandgap.

### Mechanism of dual-phase formation

**Fig. 4:**
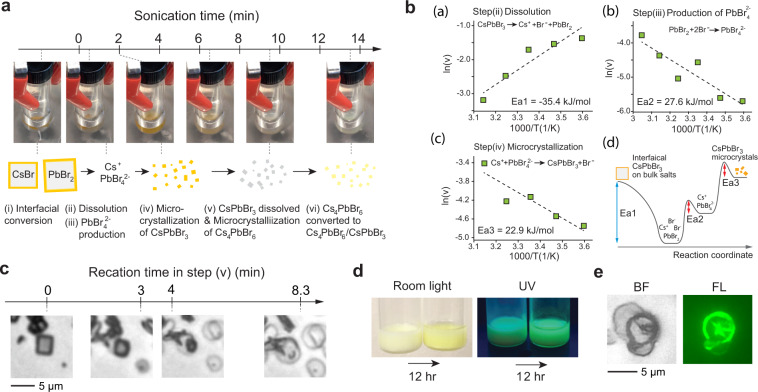
Phase transformation from single-phase CsPbBr_3_ to dual-phase Cs_4_PbBr_6_/CsPbBr_3_. **a** Time-lapse change of precursor solution (*a* = 3, *b* = 1) during ultrasonication and a schematic of six reaction intermediate steps. **b** (a–c) The temperature dependence of the reaction rate, *v* [s^−1^], for each reaction step (ii to iv); and (d) a reaction coordinate diagram of the sonochemical synthesis of CsPbBr_3_ microcrystals. **c** Bright-field images showing the phase transformation of single-phase CsPbBr_3_ cuboids to single-phase Cs_4_PbBr_6_ microdiscs. **d** The color and photoluminescence of solutions immediately after ultrasonication and after 12-h incubation under room light and UV light. **e** Bright-field and fluorescence images of dual-phase Cs_4_PbBr_6_/CsPbBr_3_ microdiscs.

To gain insights into the mechanism of dual-phase formation, we investigated distinct intermediate reaction steps, which involves color changes of the solution. The sonochemical synthesis of Cs_4_PbBr_6_/CsPbBr_3_ composite is comprised six distinct steps: the formation of orange-color CsPbBr_3_ (steps i–iv), the phase transformation from orange-color CsPbBr_3_ to white-color single-phase Cs_4_PbBr_6_ (step v), and the formation of dual-phase Cs_4_PbBr_6_/CsPbBr_3_ (step vi) (Fig. 4a; Supplementary Movie 1).

Our interpretation of the process is as follows. When CsBr and PbBr_2_ salts were mixed with DMF with concentration of *a* = 3 and *b* = 1, orange-color CsPbBr_3_ layer is immediately formed via interfacial conversion on the surface of undissolved salts [step i]. The ultrasonic pressure and temperature modulation allows the remaining salts to be completely dissolved [step ii]. Simultaneously, the reaction intermediate, ${\mathrm{PbBr}}_4^{2 - }$, increases via:

$${\mathrm{PbBr}}_2\left( {\mathrm{s}} \right) + 2{\mathrm{Br}}^-({\mathrm{sol}}) \to {\mathrm{PbBr}}_4^{2 - }({\mathrm{sol}})[{\mathrm{step}}\,{\mathrm{iii}}]$$

Since the reaction species are optically transparent, the solution turns clear. The spontaneous nucleation and growth of CsPbBr_3_ occur when the concentration of ${\mathrm{PbBr}}_4^{2 - }$ reaches the level of saturation, at which the solution turns orange. The crystallization reaction may be described as:

$${\mathrm{Cs}}^ + + {\mathrm{PbBr}}_4^{2 - } \to {\mathrm{CsPbBr}}_3\left( {\mathrm{s}} \right) + {\mathrm{Br}}^ - \left( {{\mathrm{sol}}} \right)\left[ {{\mathrm{step}}\,{\mathrm{iv}}} \right]$$

We measured the time trace of the color intensity (steps i–iv) (Supplementary Fig. 19), and calculated the reaction rates from the slope and duration of the color intensity profile. Figure 4b shows logarithmic plots of the reaction rates, *v*, as a function of the reciprocal of temperature *T*, and an overall reaction coordinate diagram. From the curve fitting of the data with the Arrhenius and Eyring equation, we obtained *E*_*a*_ = −35 kJ/mol for step (ii), 27 kJ/mol for step (iii), and 23 kJ/mol for step (iv). The negative activation energy and exothermic dissolution of bulk CsPbBr_3_ in step (ii) are due to low lattice-formation energy of CsPbBr_3_. This low energy barrier is a double-edged sword making LHPs easy to be crystallized and degraded^22^. The measured activation energies of the step (iii) and step (iv) are approximately three times smaller than that of conventional thin film formation (86 kJ/mol)^23^. The reduced activation energy likely comes from the vibrant oscillation of the pressure and temperature in ultrasonication microbubbles^24^. This low activation energy is a key to the rapid synthesis.

To understand the transformation from intermediate single-phase CsPbBr_3_ to single-phase Cs_4_PbBr_6_, we stopped ultrasonication after 10 min right after step (iv), transferred a titer amount of the solution onto a glass substrate and examined the sample using bright-field optical microscopy (Fig. 4c). Under the microscope, we observed that Cs_4_PbBr_6_ microdiscs appeared as CsPbBr_3_ microcuboids were dissolving (Supplementary Movie 2). The formation of Cs_4_PbBr_6_ can be described as:

$${\mathrm{PbBr}}_4^{2 - }\left( {{\mathrm{sol}}} \right) + 2{\mathrm{Br}}^-\left( {{\mathrm{sol}}} \right) \to {\mathrm{PbBr}}_6^{4 - }\left( {{\mathrm{sol}}} \right)$$

$${\mathrm{PbBr}}_6^{4 - }\left( {{\mathrm{sol}}} \right) + 2{\mathrm{Cs}}^ + \left( {{\mathrm{sol}}} \right) \to {\mathrm{Cs}}_4{\mathrm{PbBr}}_6\left( {\mathrm{s}} \right)$$

The fluorescence quantum yield of the microdiscs is nearly zero immediately after their formation, but gradually increases over time (Fig. 4d). This is due to the conversion of single-phase Cs_4_PbBr_6_ to dual-phase Cs_4_PbBr_6_/CsPbBr_3_ (step vi). This conversion occurs spontaneously at room temperature, but at a much slower speed over 12 h. The final Cs_4_PbBr_6_/CsPbBr_3_ microdiscs emit bright fluorescence (Fig. 4e). The conversion of single-phase Cs_4_PbBr_6_ microdiscs proceeds with slow self-formation of CsPbBr_3_ NCs in the Cs_4_PbBr_6_ matrix, releasing CsBr to the solution, via:

$${\mathrm{Cs}}_4{\mathrm{PbBr}}_6\left( {\mathrm{s}} \right) \to \left( {1 - x} \right){\mathrm{Cs}}_4{\mathrm{PbBr}}_6/x{\mathrm{CsPbBr}}_3\left( {\mathrm{s}} \right) + 3x{\mathrm{CsBr}}\left( {{\mathrm{sol}}} \right)$$

where *x* («1) denotes the amount of conversion. A similar CsBr extraction process has previously been observed during the evolution of single-phase Cs_4_PbBr_6_ to single-phase ${\mathrm{CsPbBr}}_{3}$^7,25^.

The sonochemical synthesis of dual-phase CsPb_2_Br_5_/CsPbBr_3_ composite (*a* = 1, *b* = 3) appeared to be straightforward without producing any apparent intermediates. Time-lapse video (Supplementary Fig. 20, Supplementary Movie 3) shows that as soon as the precursor salts are placed in DMF, orange-color CsPbBr_3_ layers are formed on the surface of the salts. After 2 min of ultrasonication, the entire solution turns yellow as CsPb_2_Br_5_/CsPbBr_3_ microparticles are produced. This fast and simple formation is in contrast to the slow formation of Cs_4_PbBr_6_/CsPbBr_3_. Considering the tetragonal structure of CsPb_2_Br_5_ with alternating Cs^+^ and ${\mathrm{Pb}}_2{\mathrm{Br}}_5^ -$ layers, the formation mechanism may be described as:

$$2{\mathrm{PbBr}}_2\left( {\mathrm{s}} \right) + {\mathrm{Br}}^ - \left( {{\mathrm{sol}}} \right) \to {\mathrm{Pb}}_2{\mathrm{Br}}_5^ - \left( {{\mathrm{sol}}} \right)$$

$${\mathrm{Pb}}_2{\mathrm{Br}}_5^ - \left( {{\mathrm{sol}}} \right) + {\mathrm{Cs}}^ + \left( {{\mathrm{sol}}} \right) \to {\mathrm{CsPb}}_2{\mathrm{Br}}_5\left( {\mathrm{s}} \right)$$

During the growth of CsPb_2_Br_5_, the self-formation of CsPbBr_3_ NCs in the CsPb_2_Br_5_ matrix simultaneously occurs by releasing PbBr_2_ to the solution:

$${\mathrm{CsPb}}_2{\mathrm{Br}}_5\left( {\mathrm{s}} \right) \to \left( {1 - x} \right){\mathrm{CsPb}}_2{\mathrm{Br}}_5/x{\mathrm{CsPbBr}}_{\mathrm{3}}\left( {\mathrm{s}} \right) + x{\mathrm{PbBr}}_2\left( {{\mathrm{sol}}} \right).$$

The release of PbBr_2_ during the self-formation of NCs promotes the formation of the CsPb_2_Br_5_ matrix.

In summary, we have shown that the sonochemical processes led to rapid synthesis of dual-phase perovskites. Both Cs_4_PbBr_6_/CsPbBr_3_ and CsPb_2_Br_5_/CsPbBr_3_ composites have well-defined endotaxy structures with good lattice matching between embedded CsPbBr_3_ NCs and the non-luminescent matrices^4,9,26^. The high solid-state PLQY of >40% in Cs_4_PbBr_6_/CsPbBr_3_ and efficient lasing from single CsPbBr_3_ microparticles as small as 2 µm attest the high quality of the microcrystals. Lastly, our real-time measurement data suggest that CsPbBr_3_ NCs in the Cs_4_PbBr_6_ or CsPb_2_Br_5_ matrix is formed via a partial extraction of CsBr or PbBr_2_. Single- and dual-phase CsPbBr_3_-based microparticles may prove to be useful building blocks for optical devices.

## Methods

### Chemicals and reagents

CsBr (99.99%), PbBr_2_ (99.99%), N,N-dimethylformamide (anhydrous, 99.8%), acetone (99.9%), ethyl acetate (EtoAC) (anhydrous, 99.8%), γ-butyrolactone (GBL) (99.9%), and dimethyl sulfoxide (DMSO) (anhydrous, 99.9%) were purchased from Sigma-Aldrich. All reagents were used as received from Sigma-Aldrich without further purification.

### Sonochemical synthesis of single-phase perovskite microcrystals

For producing inorganic perovskite CsPbBr_3_, CsBr and PbBr_2 _were dispersed at an equal concentration in 1 mL of N,N-dimethylformamide (DMF) in a vial. The typical concentration was 0.075 M (i.e., *a* = 1 and *b* = 1) or its multiples up to 0.3 M (*a* = *b* = 2, 3, or 4). The vial was placed into a bath-type ultrasonicator (Elmasonic P60H, Elma) or a single-step tip ultrasonicator (Fisherbrand Q125) in room temperature and irradiated with ultrasonic waves (frequency: 20 kHz∼80 kHz). After 2–3 min of ultrasonication, single-phase CsPbBr_3_ microcrystals were spontaneously crystallized and dispersed in the solution.

### Sonochemical synthesis of dual-phase Cs_4_PbBr_6_/CsPbBr_3_ microcrystals

Ultrasonication of 1 mL of DMF solution of CsBr (0.225 M or 0.3 M) and PbBr_2_ (0.075 M) for 2 min yields single-phase CsPbBr_3_ microcrystals. Continuing ultrasonication for additional several min makes the orange-colored solution to white, opaque dispersion of single-phase Cs_4_PbBr_6_ microparticles, and then to lemon-colored solution of dual-phase Cs_4_PbBr_6_/CsPbBr_3_ microparticles. This process takes about 13–15 min. After the ultrasonication has been stopped, the color of the solution becomes gradually brighter at room temperature overnight. The morphologies of the final particles are a mixture of hexagon and rhombus. As an alternative way to synthesize dual-phase Cs_4_PbBr_6_/CsPbBr_3_ microparticles, after the synthesis of CsPbBr_3_ microcrystals by 2 min of ultrasonication, the solution is removed from the ultrasonicator, and then vigorous shaking is applied for 1 h until the color turns to light green. Likewise, the lemon color becomes gradually intense over time in room temperature.

### Sonochemical synthesis of single-phase Cs_4_PbBr_6_ microcrystals

For single-phase Cs_4_PbBr_6_, CsBr (0.075 M), and PbBr_2_ (0.0375 M) in 1 mL of DMF were used as a precursor solution. In the case of CsBr (0.15 M) and PbBr_2_ (0.075 M), a mixture of single-phase CsPbBr_3_ particles and Cs_4_PbBr_6_ particles were formed.

### Sonochemical synthesis of dual-phase CsPb_2_Br_5_/CsPbBr_3_ microcrystals

Dual-phase CsPb_2_Br_5_/CsPbBr_3_ were obtained when the concentration of PbBr_2_ was higher than the concentration of CsBr. Regular truncated octahedron morphology was obtained with CsBr (0.075 M) and PbBr_2_ (0.15 M). Cuboctahedron particles were obtained with CsBr (0.075 M) and PbBr_2_ (0.225 M). For wedding cake or fibrous structure with rough surface, CsBr (0.075 M) and PbBr_2_ (0.3 M) were used.

### Structural characterization

For SEM and EDX measurements, LHPs microcrystals were transferred onto a chipped Si wafer by drop-casting and imaged using a Zeiss Merlin high-resolution SEM equipped with an EDX detector operated at 15 kV. For TEM measurements, samples were prepared by drop-casting LHP microparticles onto TEM grids (Ted Pella). TEM images were acquired using a FEI Tecnai Multipurpose TEM operated at 120 kV. The illumination beam was expanded to avoid sample damage. For PXRD measurements, PXRD patterns over 2*θ* angles from 10° to 60° were collected using a PANalytical X’Pert PRO high-resolution X-ray diffraction system with a CuKα irradiation source. These measurements were performed at MIT Center for Material Science and Engineering (CMSE).

### Optical characterization

See Supplementary Methods.

### Reporting summary

Further information on research design is available in the Nature Research Reporting Summary linked to this article.

## Supplementary information

Supplementary Information

Description of Additional Supplementary Files

Peer Review File

Reporting Summary

Supplementary Movie 1

Supplementary Movie 2

Supplementary Movie 3

## Data Availability

The main data supporting the finding of this study are available within the paper and its Supplementary Information file. Other relevant data are available from the corresponding author upon reasonable request.
