# Supplementary material for: Structure and optical properties of perovskite-embedded dual-phase microcrystals synthesized by sonochemistry
Source: Commun Chem. 2020 Feb 7;3:15. doi: 10.1038/s42004-020-0265-6 (PMC9814672; doi:10.1038/s42004-020-0265-6)
Supplement: Supplementary file 1 — Supplementary Information [file 42004_2020_265_MOESM1_ESM.pdf]

Supplementary Information for

Structure and optical properties of perovskite-embedded dual-phase microcrystals synthesized by sonochemistry

Sangyeon Cho[1,2] and Seok Hyun Yun[1,2*]

[1]Wellman Center for Photomedicine, Massachusetts General Hospital and Harvard Medical School, Cambridge, Massachusetts, 02139, USA

[2]Harvard-MIT Health Sciences and Technology, Massachusetts Institute of Technology, Cambridge, Massachusetts, 02139, USA

Corresponding Author
E-mail: syun@hms.harvard.edu

- Supplementary Methods ………………… pp. 2
- Supplementary Figures (1 - 20) ………………… pp. 3-16
- Supplementary Tables (1) ………………… p. 17

Supplementary Methods

Optical characterization. Optical absorbance spectra were measured using a spectrophotometer (BioTEk, Epoch2). Widefield brightfield and fluorescence images were obtained using an optical microscope (Keyence, BZ-X). For time-resolved photoluminescence measurements, we used a picosecond laser (VisIR-765, PicoQuant), which was frequency-doubled to 382 nm using a nonlinear BBO crystal (Fig. S16), a single-photon avalanche photodiodes (Micro Photonics Devices) with a response time of 50 ps, and a time-correlated single-photon counting board (TimeHarp 260, PicoQuant) with a resolution of 25 ps. For absolute quantum yield measurements, a vial of dispersed LHPs microcrystals in solution was placed in an integrating sphere (Thorlabs) and excited using the 382-nm frequency-doubled picosecond laser. The spectra of light collected from the integrating sphere was coupled, via a multimode fiber, to a spectrometer (Shamrock, Andor) consisting of a diffraction grating and an electron multiplier charge-coupled device (EMCCD) camera. For lasing experiments, LHPs microcrystals were dry-transferred onto a microscope glass slide, covered with a glass cover slip, and then sealed using UV curing adhesive while N_2 gas was flown onto the sample. The specimen was placed in a home-built epi-fluorescence microscopy setup (Fig. S16). The pump source was an optical parametric oscillator (OPO, Optotek HE 355 LD) tuned to 480 nm and circularly polarized, with a repetition rate of 20 kHz and a pulse duration of 4 ns. Using a 0.6 NA, 50x air objective lens (Nikon), the full-width-at-half-maxima (FWHM) size of the pump beam on the sample was adjusted to ~7.5 µm. The emission from the sample collected by the objective lens was passed through a dichroic mirror and a dichroic filter and split to an EMCCD camera (Luca, Andor) for wide-field imaging and to the grating-based EMCCD spectrometer (Shamrock, Andor). With an entrance slit width of 100 µm, the measurement spectral resolution was ~0.13 nm. As an excitation source for photoluminescence analysis, we used a continuous-wave, diode-pumped laser (491 nm, Cobolt Calypso). All measurements were conducted at room temperature.

Supplementary Figures

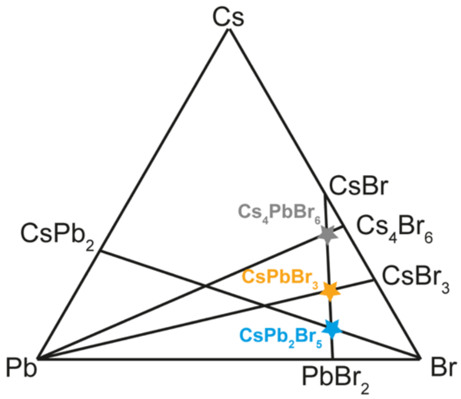

Supplementary Figure 1. Ternary diagram of Cs, Pb and Br, presenting accessible endotaxy materials in a mixed CsBr-$PbBr_2$ system.

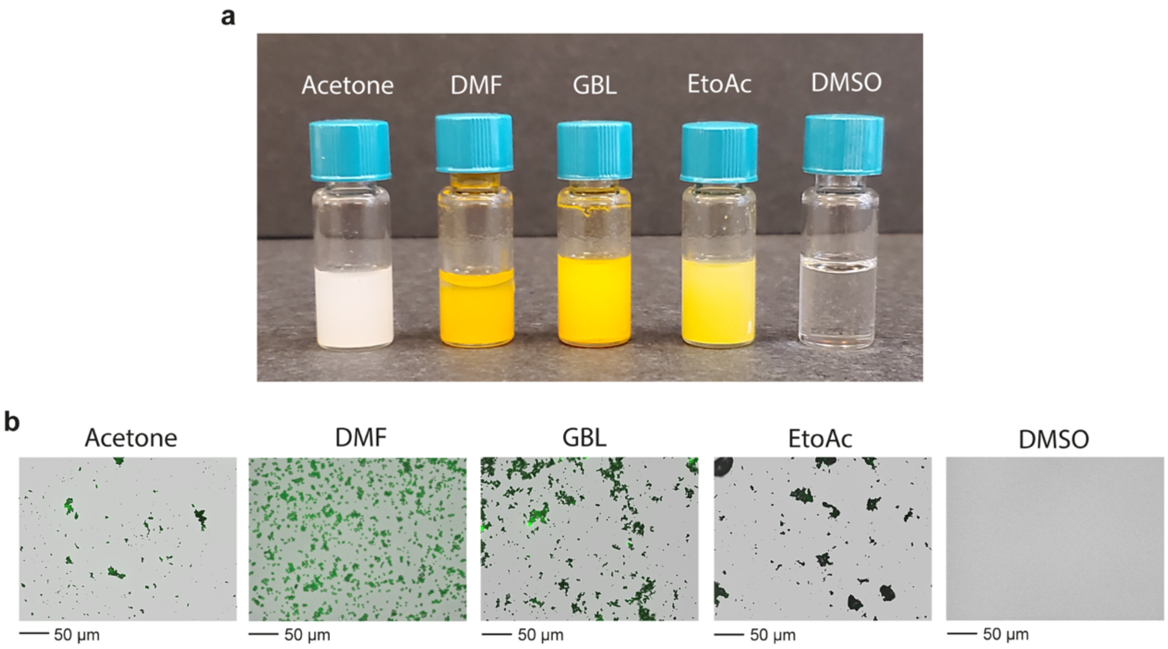

Supplementary Figure 2. Sonochemistry products obtained with different polar aprotic solvents with slightly different dipole moments (D), which include acetone (2.91D), N,N-dimethylformamide (DMF, 3.82 D), γ-butyrolactone (GBL, 4.24D), ethyl acetate (EtOAc, 1.78D), and dimethyl sulfoxide (DMSO, 3.96D). The concentration of CsBr and $PbBr_2$ was each 75 mM (a = b = 1). **a**, Photos of the reaction products obtained in different polar aprotic solvents. **b**, A green fluorescence image, overlaid with a bright-field image, of the reaction products transferred to glass slides. Only the products obtained with DMF present typical cuboidal $CsPbBr_3$ microparticles capable of emitting green fluorescence.

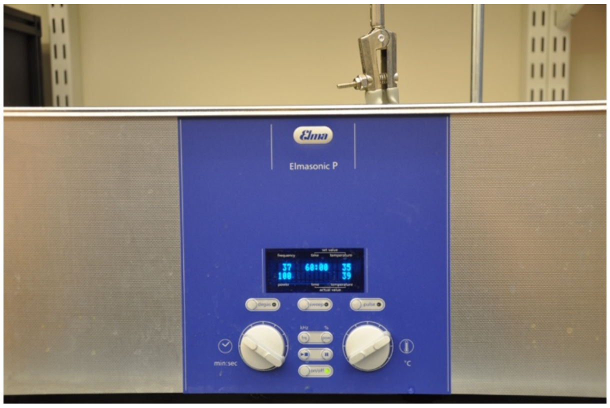

Supplementary Figure 3. A temperature-controlled water bath ultrasconicator used in the experiment (Elma, Elmasonic P60H) with a frequency/power setting of 37kHz / 180W.

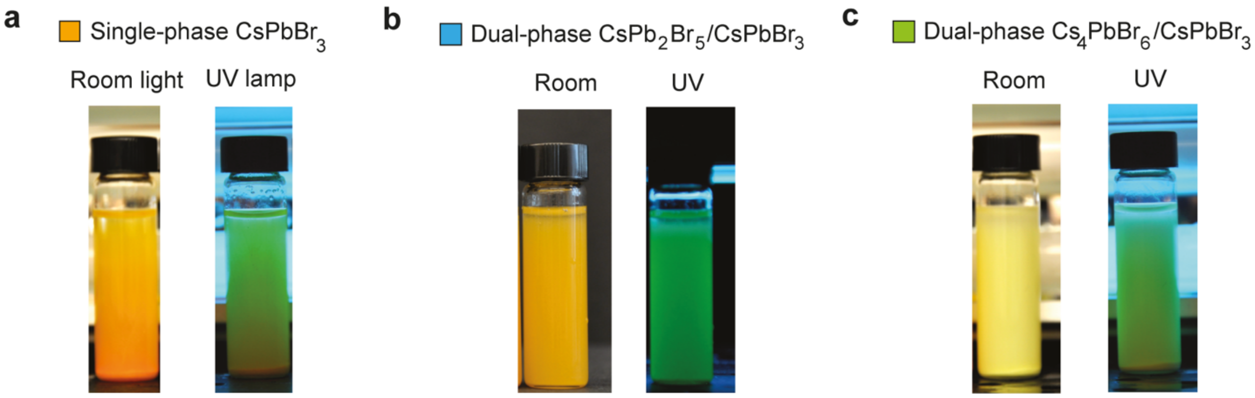

Supplementary Figure 4. Photos of various products of sonochemistry. **a**, A dispersion of $CsPbBr_3$. **b**, Dual-phase $Cs_4PbBr_6/CsPbBr_3$ after ultrasonication and overnight incubation. **c**, Dual-phase $CsPb_2Br_5/CsPbBr_3$ in DMF.

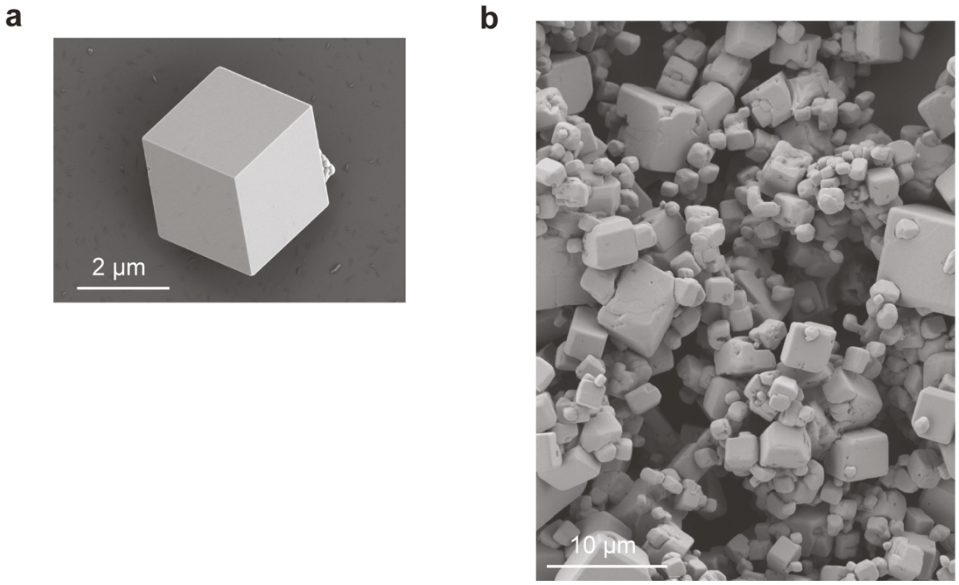

Supplementary Figure 5. SEM Images of single phase $CsPbBr_3$ microcrystals. **a**, A cuboidal particle synthesized with concentrations of a = 1 and b = 1 (75 mM). **b**, Submicron $CsPbBr_3$ crystals synthesized at higher precursor concentrations of a = b = 4 (300 mM).

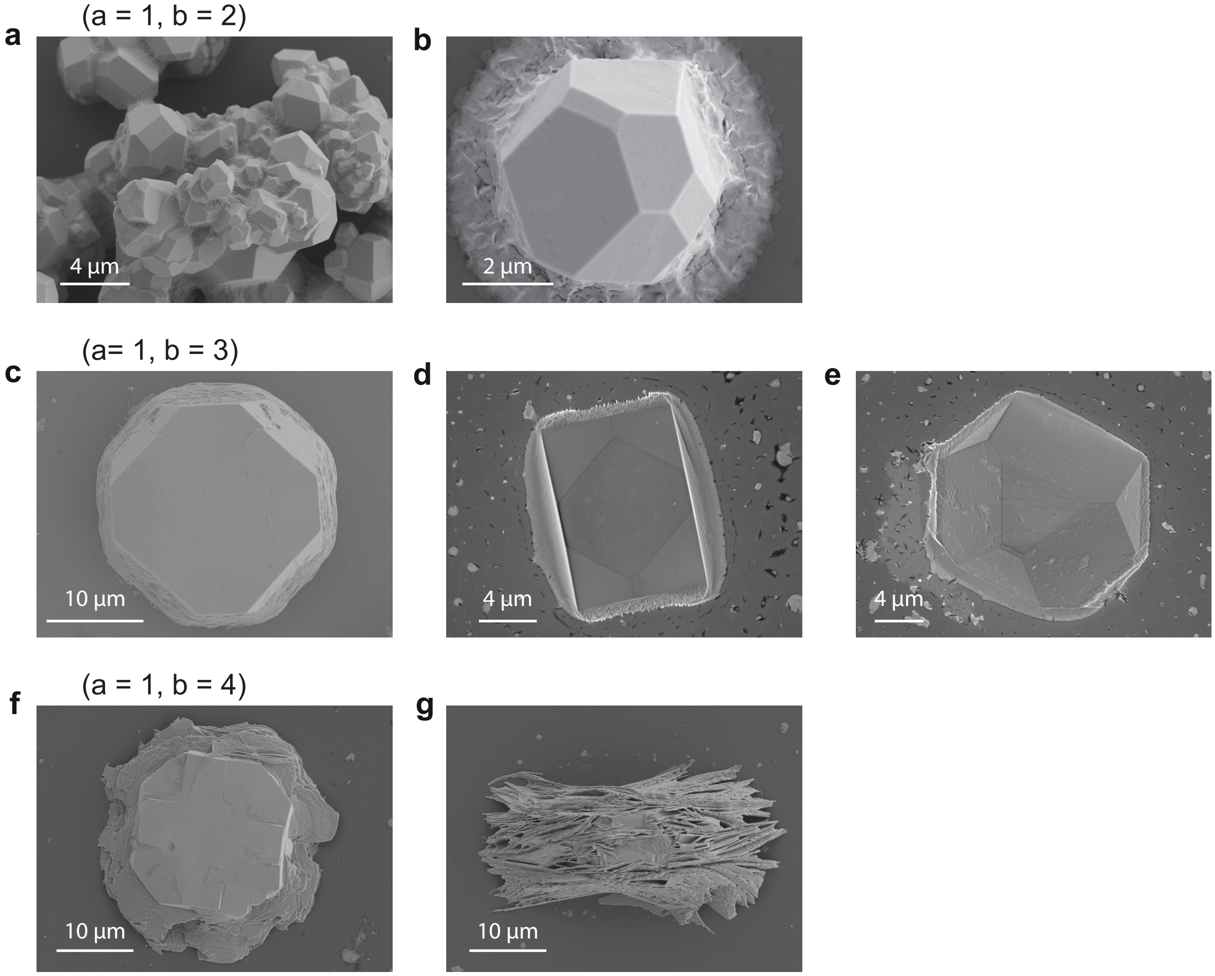

Supplementary Figure 6. SEM images of dual-phase $CsPb_2Br_5/CsPbBr_3$ microcrystals synthesized with different precursor concentrations. **a**, Aggregates of truncated octahedron crystals. **b**, Truncated octahedron crystal. (**c-e**) Cuboctahedron crystals. **f**, Wedding-cake crystal. **g**, Fibrous crystal. The concentration of CsBr was fixed at 75 mM (a = 1), and the concentration of $PbBr_2$ was 150 mM in **a**-**b**, 225 mM in **c-d**, and 300 mM in **f**-**g**.

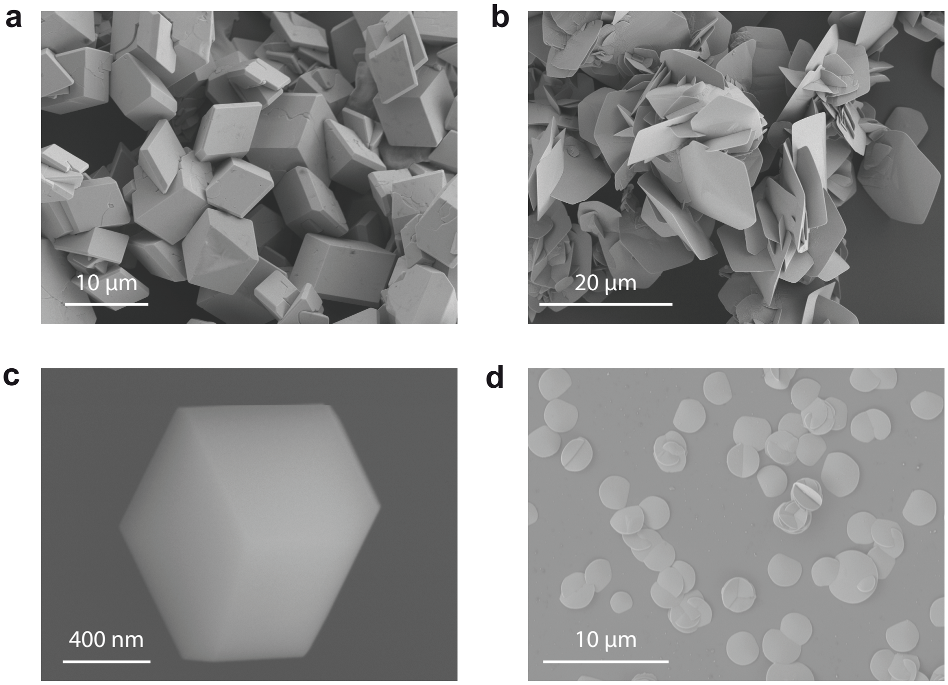

Supplementary Figure 7. SEM images of dual-phase Cs_4PbBr_3/$CsPbBr_3$ microcrystals. **a**, Rhombus crystals. **b**, Hexagonal plates. **c**, A truncated rhombus crystal. **d**, Microdisc sheets.

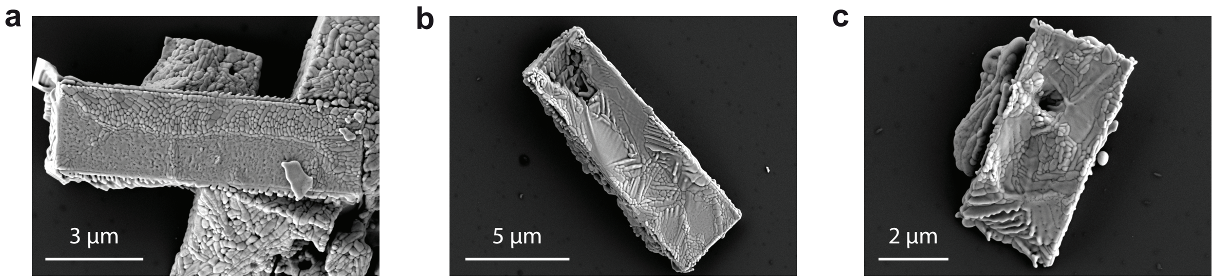

Supplementary Figure 8. SEM images of three single-phase Cs_4PbBr_6 microcrystals (**a**-**c**).

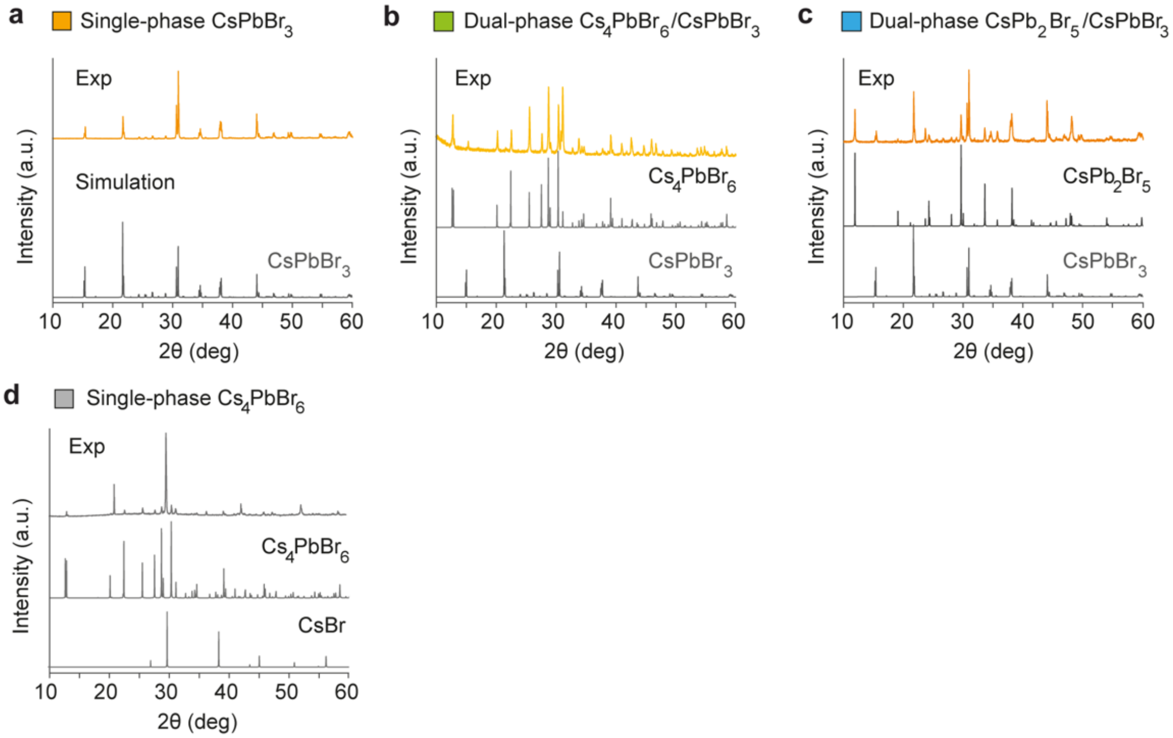

Supplementary Figure 9. Power X-ray diffraction (PXRD) patterns of various microparticles synthesized by sonochemistry. **a**, single-phase $CsPbBr_3$. **b**, $Cs_4PbBr_6/CsPbBr_3$. **c**, $CsPb_2Br_5$ / $CsPbBr_3$. **d**, Cs_4PbBr_6. Top trace in each graph: Experimental data. Grey curves: simulation results based on an orthorhombic structure of $CsPbBr_3$ (space group *Pbnm*, a = 8.20 Å, b = 8.24 Å, c = 11.74 Å), trigonal Cs_4PbBr_6 structure (*R-3c*, a = 13.73 Å, c =17.32 Å), tetragonal $CsPb_2Br_5$ structure (*I4/mcm*, a = 8.45 Å, c = 15.07 Å), and cubic CsBr structure (Fm-3m, a = 5.234 Å).

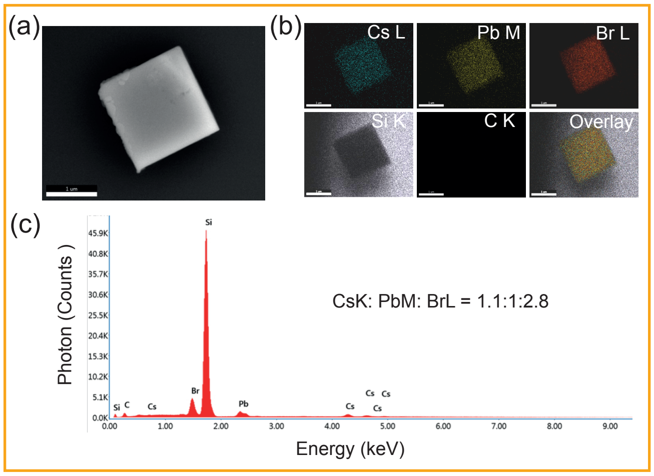

Supplementary Figure 10. Energy dispersive X-ray spectroscopy (EDS) of single-phase cuboid $CsPbBr_3$ (a=1, b=1, 75 mM). **a** Representative SEM image. **b**, Spatial elemental maps of cesium L line, lead M line, bromine L line, silicon K line, and carbon K line. **c**, Typical EDS spectrum, and the calculated stoichiometry ratio of the Cs, Pb and Br contents.

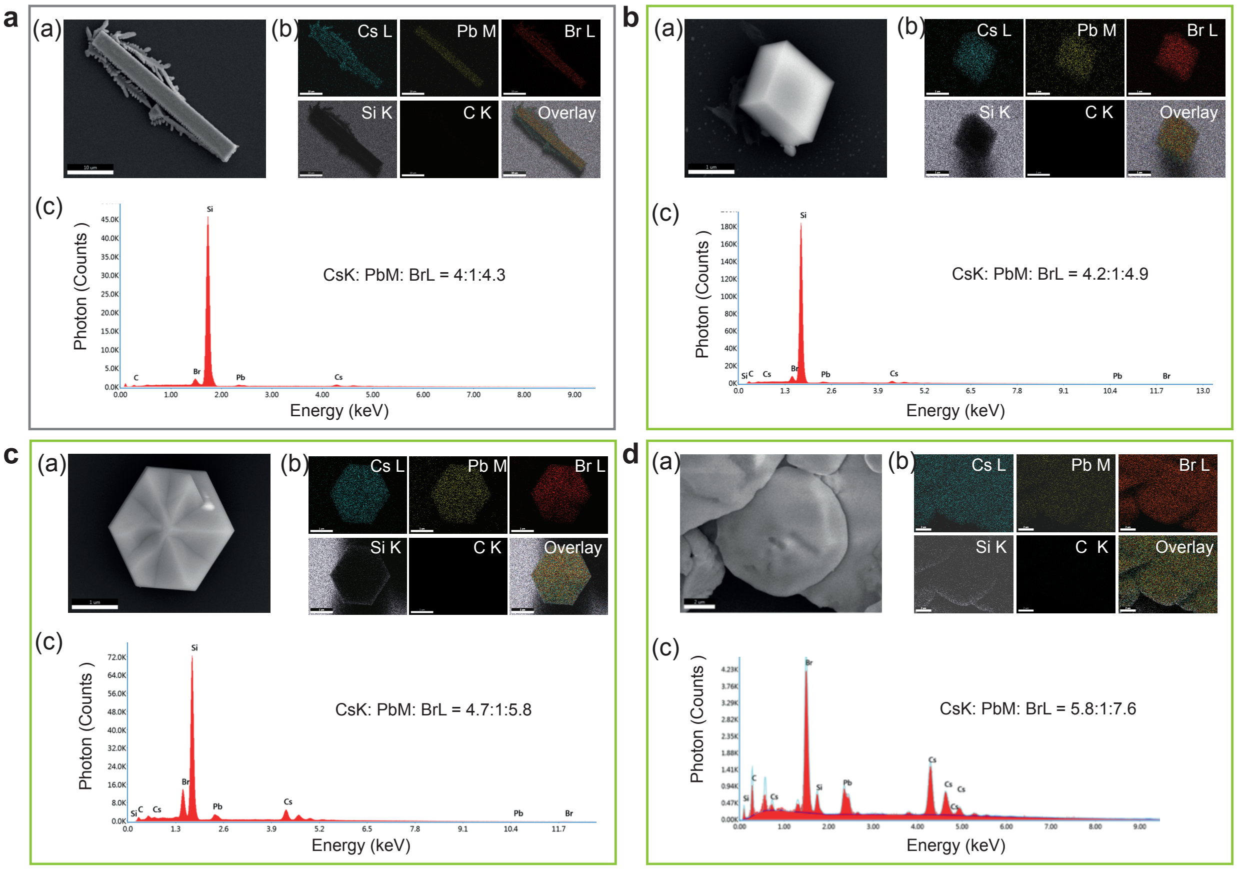

Supplementary Figure 11. EDS data of **a** single-phase granular Cs_4PbBr_6 (a=1, b=0.5), **b** dual-phase rhombus $Cs_4PbBr_6/CsPbBr_3$ (a>=2, b=1), **c** hexagonal-plate $Cs_4PbBr_6/CsPbBr_3$ (a>=2, b=1), and **d** discoidal $Cs_4PbBr_6/CsPbBr_3$ (a>=2, b=1). Each panel show (a) a representative SEM image, (b) spatial elemental maps, and (c) EDS spectrum and the calculated stoichiometry ratio of Cs, Pb and Br contents.

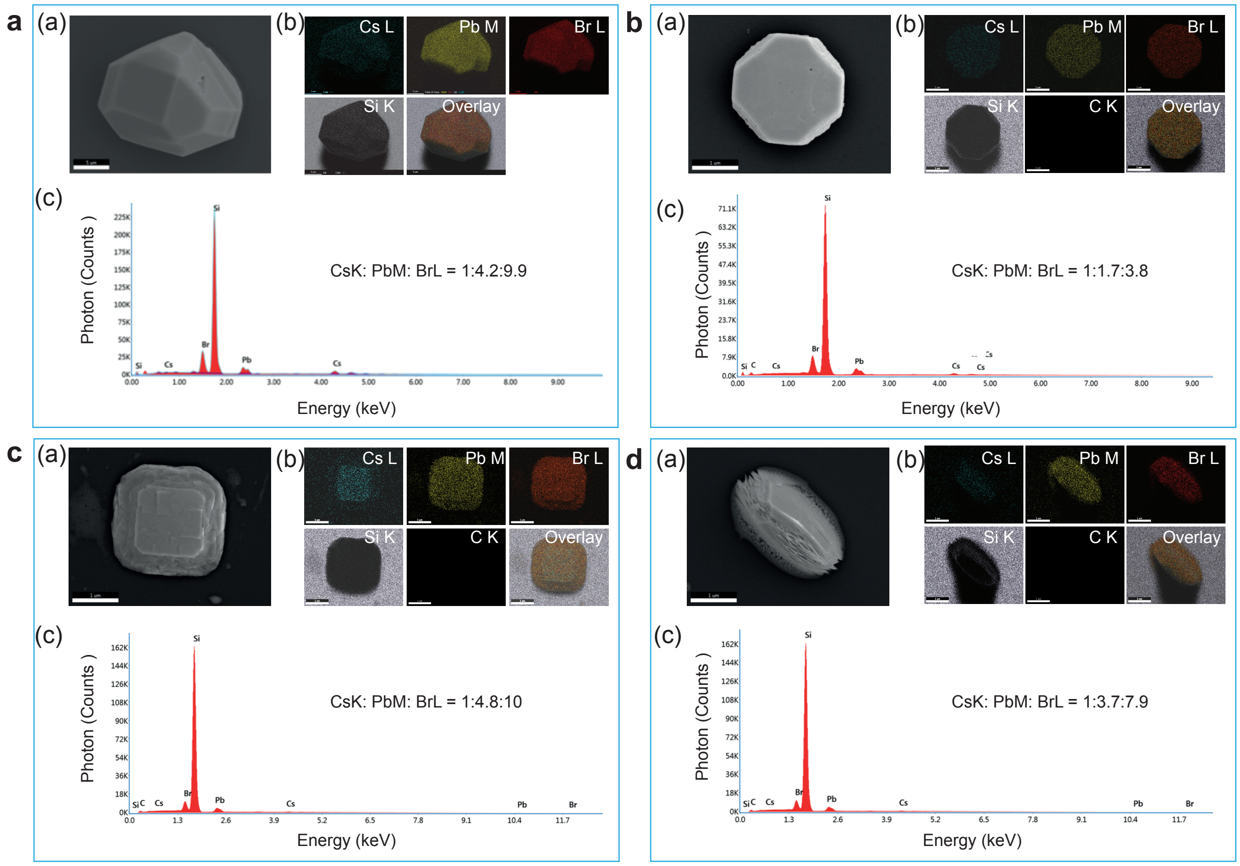

Supplementary Figure 12. EDS data of **a** dual-phase truncated octahedron $CsPb_2Br_5/CsPbBr_3$ (a=1, b=2), **b** cuboctahedron $CsPb_2Br_5/CsPbBr_3$ (a=1, b=3), **c** wedding-cake $CsPb_2Br_5/CsPbBr_3$ (a=1, b=4), and **d** fibrous $CsPb_2Br_5/CsPbBr_3$ (a=1, b=4). Each panel shows (a) a representative SEM image, (b) spatial elemental maps, and (c) EDS spectrum and the calculated stoichiometry ratio of Cs, Pb and Br contents.

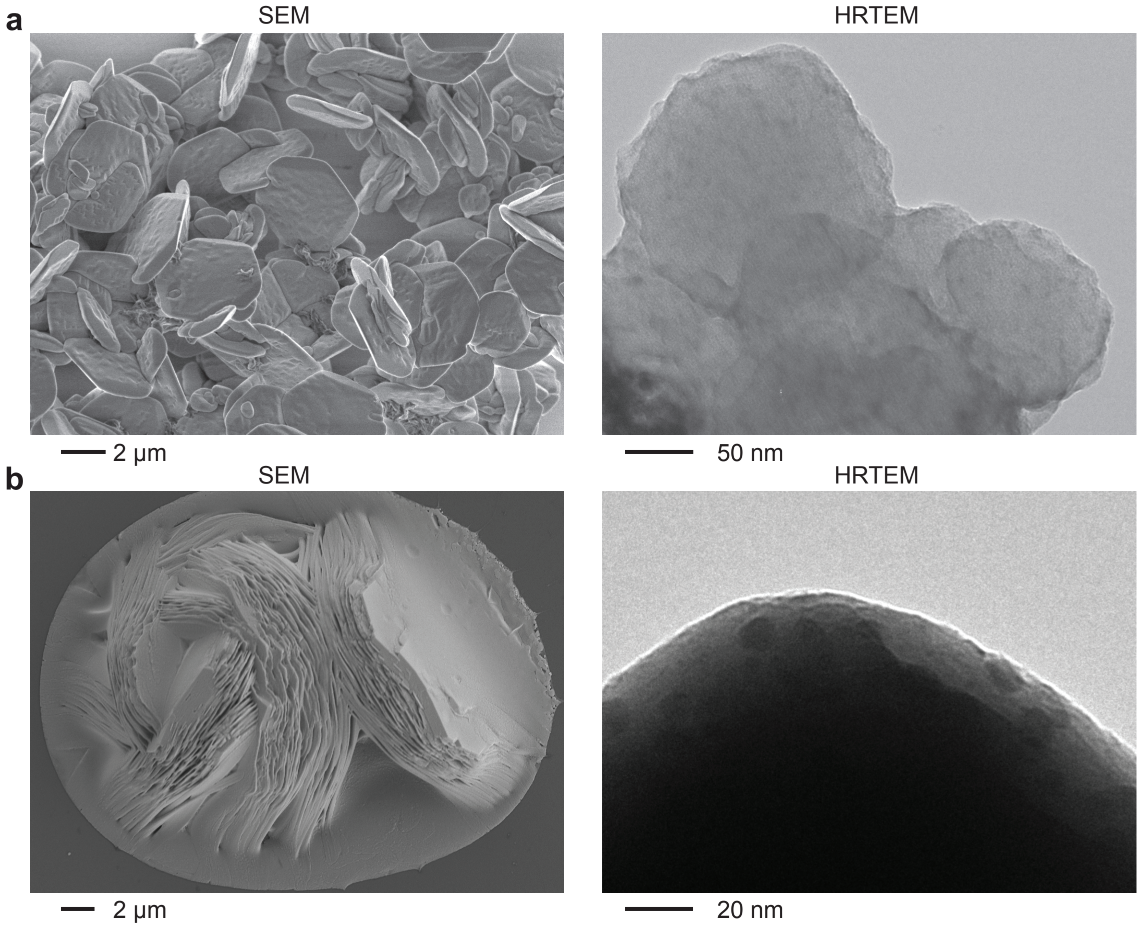

Supplementary Figure 13. SEM and HRTEM images of dual-phase microcrystals used for lattice analysis. **a**, Micro-discoidal $Cs_4PbBr_6/CsPbBr_3$ particles. **b**, Multi-sheet dual-phase $CsPb_2Br_5/CsPbBr_3$ particles.

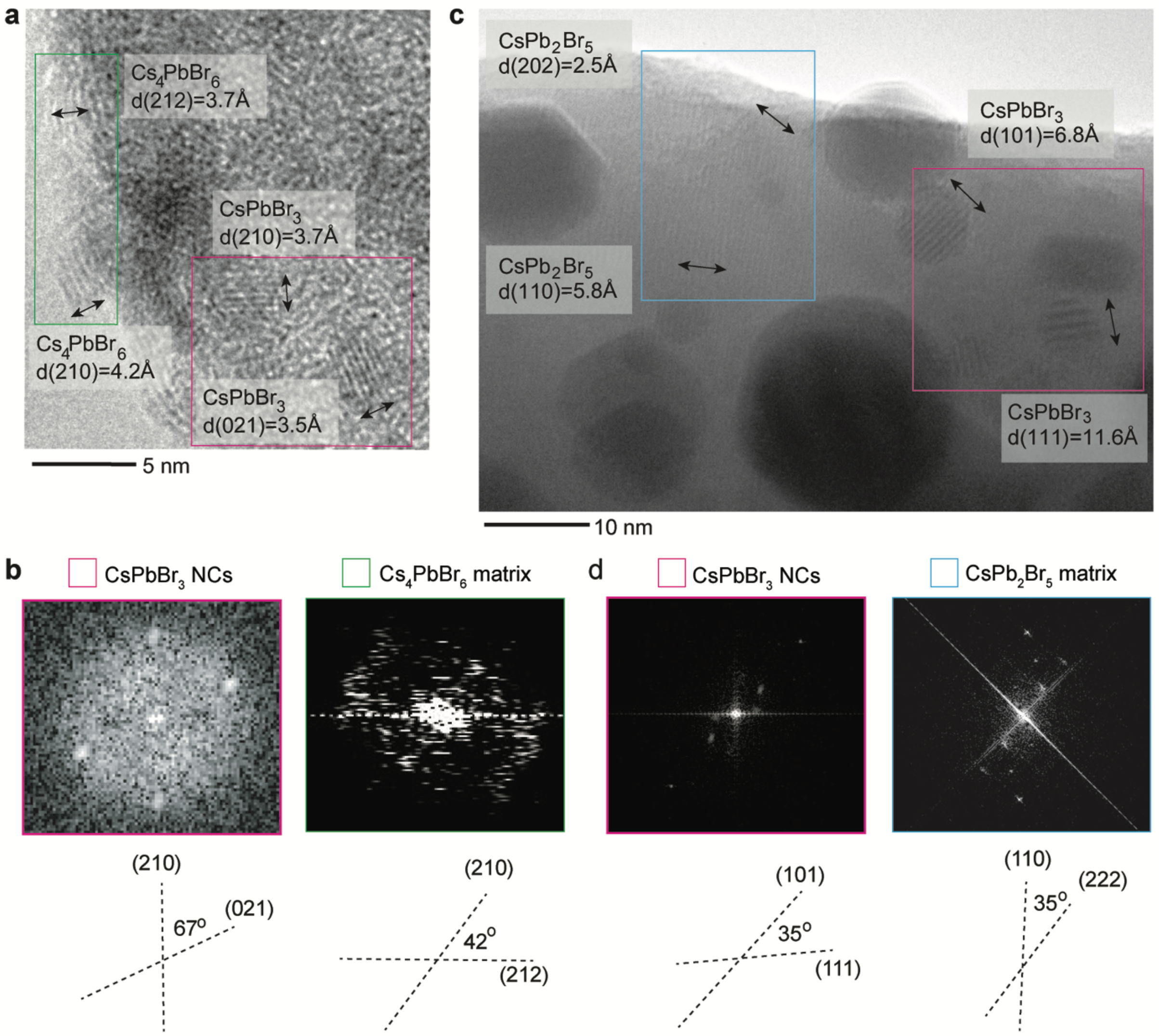

Supplementary Figure 14. HRTEM and Fourier-domain patterns. **a**-**b**, $Cs_4PbBr_6/CsPbBr_3$. **c**-**d**, $CsPb_2Br_5/CsPbBr_3$. (**a**, **c**) HRTEM images. (**b**, **d**) FFT patterns of boxed regions in (**a**, **c**) containing the lattice fringe of $CsPbBr_3$ NCs (magenta) and endotaxy matrices (green and cyan).

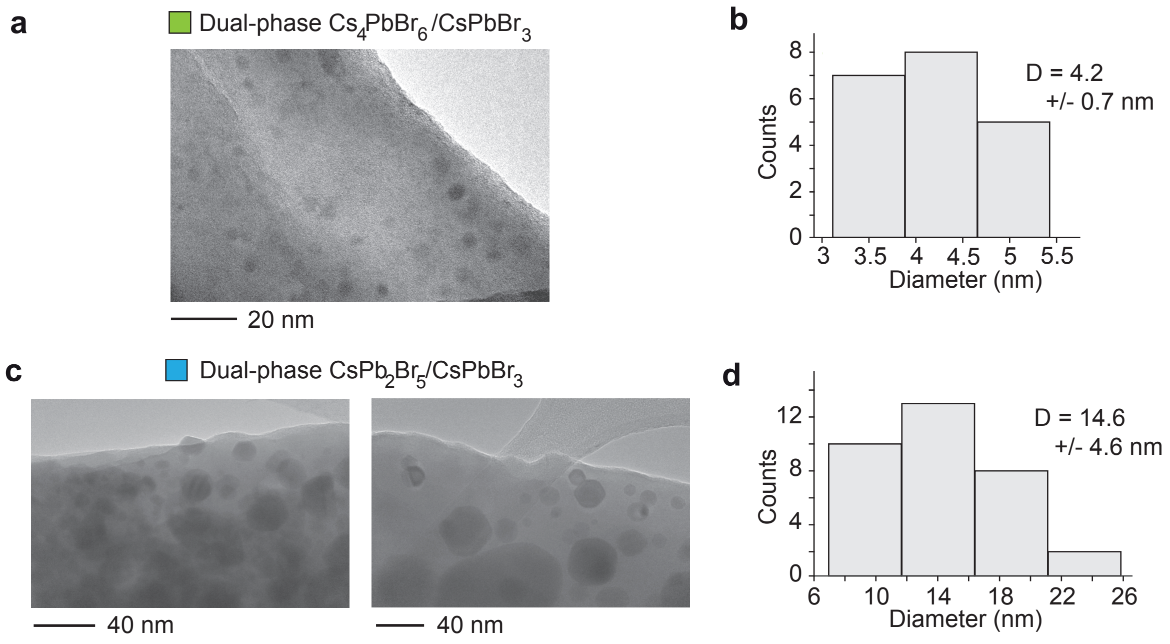

Supplementary Figure 15. TEM images of dual-phase microparticles. **a**, $Cs_4PbBr_6/CsPbBr_3$. **b**, Histogram of the measured effective diameter of $CsPbBr_3$ NCs in a Cs_4PbBr_6 matrix (N = 20, mean dia.: 4.2 nm, standard deviation: 0.7 nm). **c**, $CsPb_2Br_5/CsPbBr_3$. **d**, Histogram of the measured diameter of $CsPbBr_3$ NCs in a $CsPb_2Br_5$ matrix (N = 33, mean dia.: 14.6 nm, standard deviation: 4.6 nm)

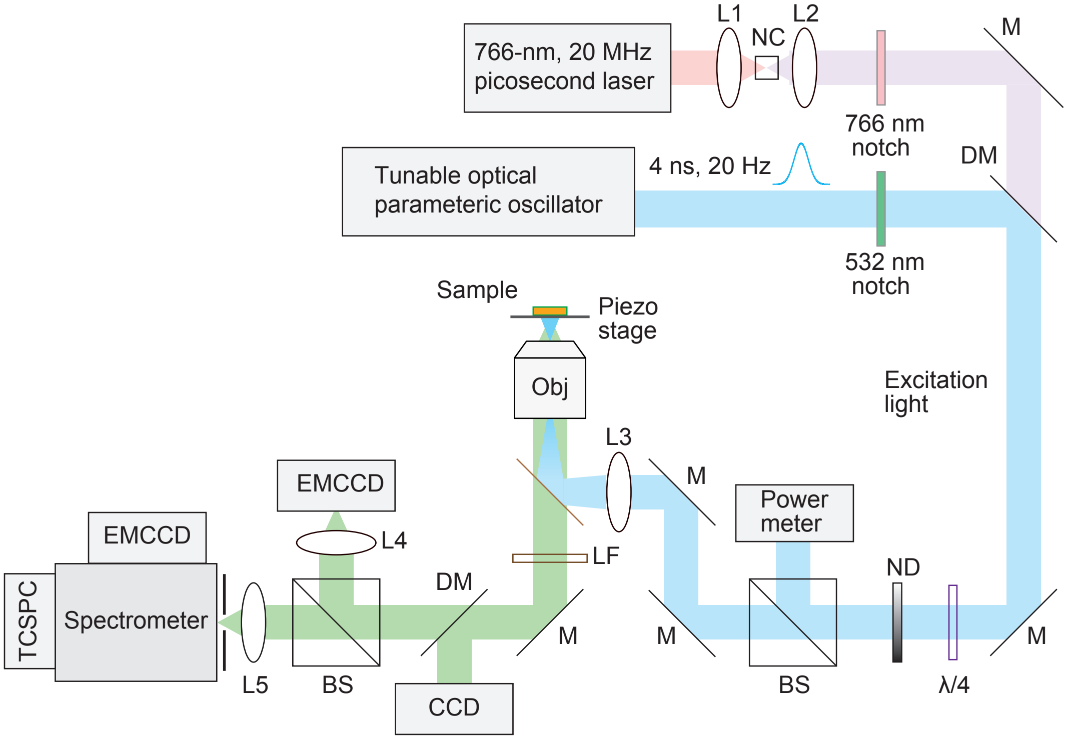

Supplementary Figure 16. Schematic of the optical measurement setup. L: lens, NC: second-harmonic generation nonlinear crystal, M: mirror, DM: dichroic mirror, ND: neutral density filter, BS: beam splitter, LF: laser line filter, CCD: charge-coupled device camera, EMCCD: electron-multiplication CCD camera, and TCSPC: time-correlation single-photon counter.

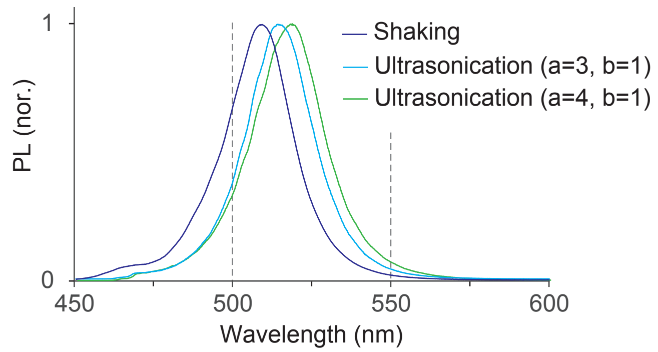

Supplementary Figure 17. Photoluminescence spectra of dual-phase $Cs_4PbBr_6/CsPbBr_3$ microcrystals synthesized at different conditions. The emission spectrum is varied from 509.5 nm to 521 nm depending on ultrasonication times and precursor concentrations. When ultrasonication is stopped in the intermediate state and followed by vigorous shaking at 800 rpm for 1 hour (purple curve), the emission center was 508.5 nm regardless of starting precursor concentrations.

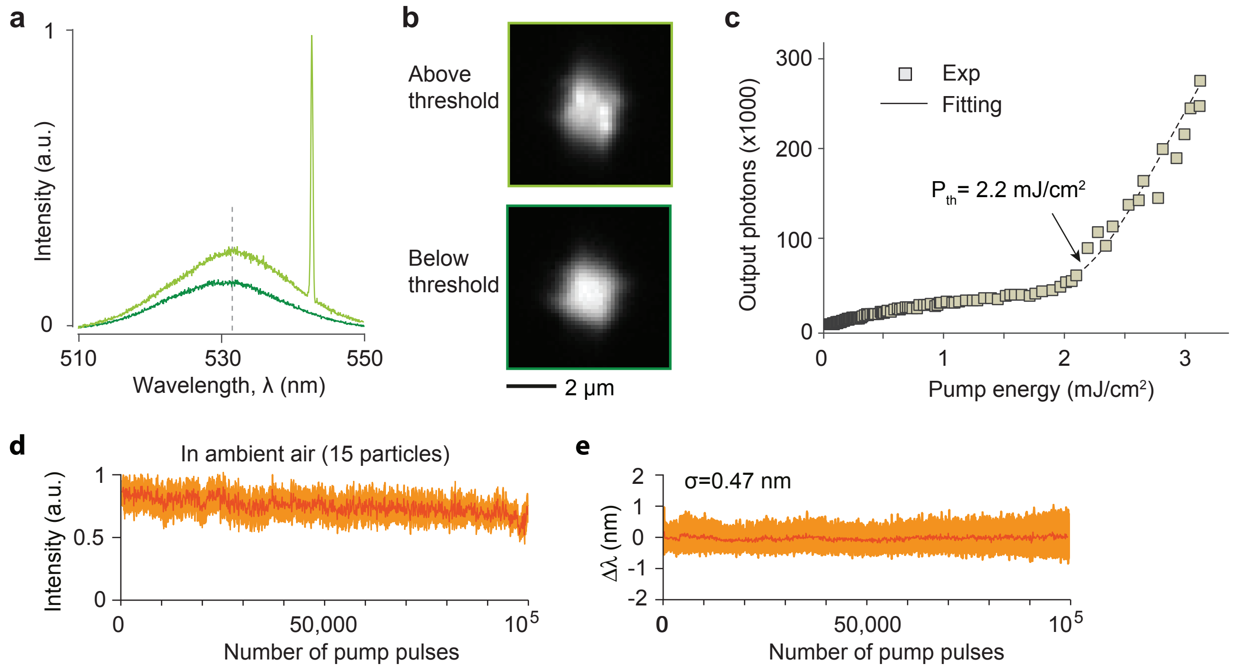

Supplementary Figure 18. a, Output spectra from a 2-µm sized $CsPbBr_3$ microcrystal upon nanosecond optical pumping (480 nm) below (light green) and above (dark green) a lasing threshold. **b**, Wide-field fluorescence images below and above the laser threshold. **c**, Light-in-light-out curve, showing a clear lasing threshold with a spontaneous emission factor (β) of ~10^{-3}. **d**-**e**, The variations of laser output intensity and lasing wavelength for bare $CsPbBr_3$ microparticles over 100,000 pump pulses at 20 Hz repetition for a duration of ~1 h (20 Hz) at $P = 2P_{th}$. Solid lines are mean values from 15 samples, and shaded regions are 95% confidence intervals of the data.

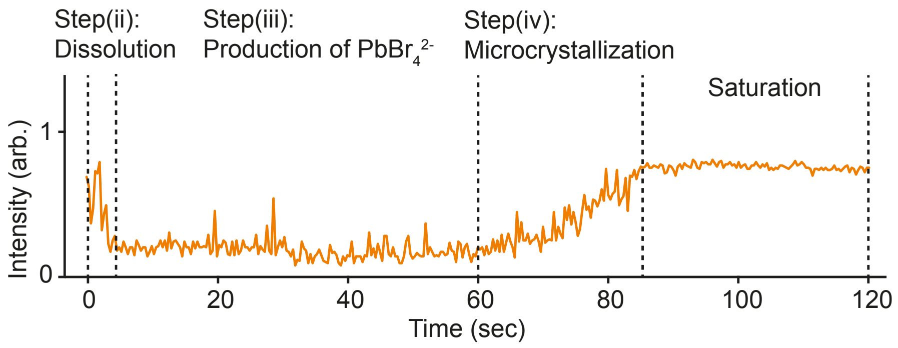

Supplementary Figure 19. The color intensity changes of a precursor solution (a = 1, b = 1, 75 mM) at 25 °C during ultrasonication that started at t = 0.

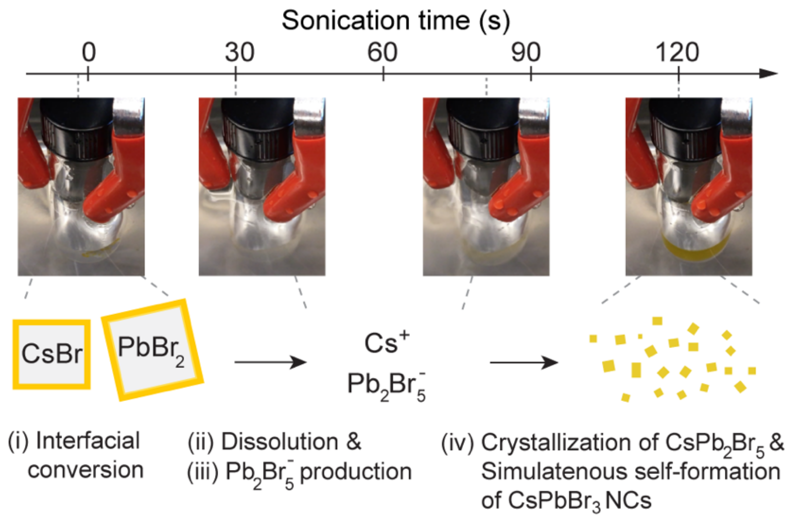

Supplementary Figure 20. Sonochemical synthesis of dual-phase $CsPb_2Br_5$/$CsPbBr_3$ microparticles. Video frames showing the color changes of a precursor solution (a = 1, b = 3) during four reaction steps (i-iv).

Supplementary Table

Supplementary Table 1. PLQY and photoluminescence decay parameters for various LHP microparticles.

	Single phase $CsPbBr_3$	$CsPbBr_3$ NCs in $CsPb_2Br_5$	$CsPbBr_3$ NCs in Cs_4PbBr_6
Measured PLQY	1.5%	0.16%	43%
τ_1 from TCSPC	0.85 ns	1.5 ns	1.2 ns
τ_2 from TCSPC	6.6 ns	5.7 ns	4.1 ns
τ_3 from TCSPC	30 ns	13 ns	17 ns
A_1 from TCSPC	0.27	0.26	0.46
A_2 from TCSPC	0.18	0.40	0.43
A_3 from TCSPC	0.55	0.34	0.11
Computed τ_{tot}	18 ns	7.0 ns	4.2 ns
Computed τ_{rad}	1200 ns	4400 ns	9.7 ns
