# Supplementary material for: Structure and optical properties of perovskite-embedded dual-phase microcrystals synthesized by sonochemistry
Source: Commun Chem. 2020 Feb 7;3:15. doi: 10.1038/s42004-020-0265-6 (PMC9814672; doi:10.1038/s42004-020-0265-6)
Supplement: Supplementary file 2 — Description of Additional Supplementary Files [file 42004_2020_265_MOESM2_ESM.pdf]

Description of Additional Supplementary Files

File Name: Supplementary Movie 1
Description: Sonochemical synthesis of dual-phase Cs_4PbBr_6/$CsPbBr_3$ microcrystals.

File Name: Supplementary Movie 2
Description: Time-lapse bright-field imaging of a conversion process from single-phase $CsPbBr_3$ microcuboid to single-phase Cs_4PbBr_6 microdisc.

File Name: Supplementary Movie 3
Description: Sonochemical synthesis of dual-phase $CsPb_2Br_5$/$CsPbBr_3$ microcrystals
